# Shallow Whole-Genome Sequencing of *Aedes japonicus* and *Aedes koreicus* from Italy and an Updated Picture of Their Evolution Based on Mitogenomics and Barcoding

**DOI:** 10.3390/insects14120904

**Published:** 2023-11-23

**Authors:** Nicola Zadra, Alessia Tatti, Andrea Silverj, Riccardo Piccinno, Julien Devilliers, Clifton Lewis, Daniele Arnoldi, Fabrizio Montarsi, Paula Escuer, Giuseppe Fusco, Veronica De Sanctis, Roberto Feuda, Alejandro Sánchez-Gracia, Annapaola Rizzoli, Omar Rota-Stabelli

**Affiliations:** 1 Center Agriculture Food Environment (C3A), University of Trento, 38010 San Michele all’Adige, Italy; nicola.zadra@fmach.it (N.Z.); alessia.tatti@fmach.it (A.T.); andrea.silverj@unitn.it (A.S.); riccardo.piccinno@unipv.it (R.P.); 2 CIBIO Department, University of Trento, 38123 Trento, Italy; veronica.desanctis@unitn.it; 3 Research and Innovation Centre, Fondazione Edmund Mach, 38010 San Michele all’Adige, Italy; daniele.arnoldi@fmach.it (D.A.); annapaola.rizzoli@fmach.it (A.R.); 4 Department of Biology, University of Padova, 35121 Padova, Italy; giuseppe.fusco@unipd.it; 5 University School for Advanced Studies IUSS Pavia, 27100 Pavia, Italy; 6 Department of Biology and Biotechnology “L. Spallanzani”, University of Pavia, 27100 Pavia, Italy; 7 Department of Genetics and Genome Biology, University of Leicester, Leicester LE1 7RH, UK; jsad1@le.ac.uk (J.D.); cl486@le.ac.uk (C.L.); rf190@leicester.ac.uk (R.F.); 8 Istituto Zooprofilattico Sperimentale Delle Venezie, 35020 Legnaro, Italy; fmontarsi@izsvenezie.it; 9 Departament de Genètica, Microbiologia i Estadística, Universitat de Barcelona, 08028 Barcelona, Spain; p.escuer@ub.edu (P.E.); elsanchez@ub.edu (A.S.-G.); 10 Institut de Recerca de la Biodiversitat (IRBio), Universitat de Barcelona, 08007 Barcelona, Spain

**Keywords:** Aedini, *Aedes koreicus*, *Aedes japonicus*, phylogeny, divergence, mitochondrial genome, invasive species, genome skimming

## Abstract

**Simple Summary:**

*Aedes* mosquitoes have been introduced into many non-native territories. These insects are often the main vectors of arboviruses such as Zika virus, West Nile virus, and Chikungunya virus. Two related species, *Aedes japonicus* and *Aedes koreicus*, originally native to East Asia, are currently expanding their range into Central and Eastern Europe. Studying these two invasive species is critical for their effective management, but there is still a lack of genome-scaled data. Here, we present the results of shallow genome sequencing of these two species. We used these data for downstream phylogenetic and barcode analyses. Our findings provide new insights into the genomics, evolution, and taxonomy of *Aedes japonicus* and *Aedes koreicus*.

**Abstract:**

*Aedes japonicus* and *Aedes koreicus* are two invasive mosquitoes native to East Asia that are quickly establishing in temperate regions of Europe. Both species are vectors of arboviruses, but we currently lack a clear understanding of their evolution. Here, we present new short-read, shallow genome sequencing of *A. japonicus* and *A. koreicus* individuals from northern Italy, which we used for downstream phylogenetic and barcode analyses. We explored associated microbial DNA and found high occurrences of *Delftia* bacteria in both samples, but neither *Asaia* nor *Wolbachia.* We then assembled complete mitogenomes and used these data to infer divergence times estimating the split of *A. japonicus* from *A. koreicus* in the Oligocene, which was more recent than that previously reported using mitochondrial markers. We recover a younger age for most other nodes within Aedini and other Culicidae. COI barcoding and phylogenetic analyses indicate that *A. japonicus yaeyamensis*, *A. japonicus amamiensis*, and the two *A. koreicus* sampled from Europe should be considered as separate species within a monophyletic species complex. Our studies further clarify the evolution of *A. japonicus* and *A. koreicus,* and indicate the need to obtain whole-genome data from putative species in order to disentangle their complex patterns of evolution.

## 1. Introduction

Aided by global trade, increased travelling, and global warming, various Aedini mosquitoes competent for arboviruses are becoming endemic in Europe, North America, and other temperate regions. As a consequence, diseases that were previously only tropical are now arising in these invaded territories, posing public health concerns [1,2,3]. Because of their capability to spread several arboviruses (such as Dengue virus, Zika virus, and Yellow fever virus) and adapt to urban environments outside their native Asian range, *Aedes aegypti* Linnaeus, 1762, and *Aedes albopictus* Skuse, 1894, quickly became the most studied species within the Aedini group [4,5].

**Figure 1:**
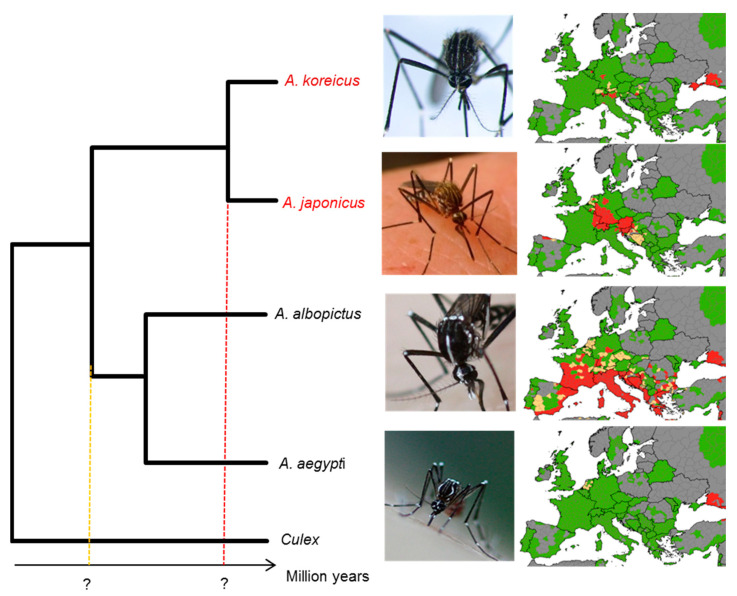
Phylogeny and European distribution of four *Aedes* species. A phylogeny of four *Aedes* mosquitoes; divergence times of *A. koreicus* and *A. japonicus* are still disputed. On the right of species names, the respective distribution in Europe is shown. The coloured species represent the species sequenced for this work. The question marks on the x-axes represent the interesting diverging nodes that we want to investigate. Their dating is still disputed. All photos are licensed under a Creative Commons license; *A. japonicus*: cydno, as CC BYNC; *A. koreicus* and *A. albopictus*: Capelli CC BY 2.0; *A. aegypti*: Monica R, CC BY 2.0. Maps have been taken and modified from the European Centre for Disease Prevention and Control, updated in February 2023 (https://www.ecdc.europa.eu/, accessed on 28 October 2022). Green: not present; Red: present; yellow: uncertain. Photo credits: (https://www.ecdc.europa.eu/, accessed on 28 October 2022).

Many other *Aedes* species are competent for arboviruses [6,7,8], but their impact on public health is much lower than that of *A. aegypti* and *A. albopictus*. This is for several reasons: Many other *Aedes* mosquitoes are confined to specific regions and have not spread globally, even if they can sustain local outbreaks [6,9,10,11]. Moreover, other *Aedes* vectors are less-efficient vectors of arboviruses or have been introduced to new territories only recently [12]. Among these, *Aedes japonicus* Theobald, 1901, and *Aedes koreicus* Edwards, 1917, are two invasive species originally from temperate East Asian regions. *Aedes japonicus* is now well established in eastern North America [13,14] and in Central Europe, and it is currently expanding its range to northern Italy [15,16]. *Aedes koreicus* is present in Belgium, Germany, Central and Eastern European countries, and is currently quickly extending its range in Italy as well (Figure 1) [17,18]. Like most other *Aedes*, these invasive species show anthropophilic behaviour and are competent vectors of arboviruses such as West Nile virus, Zika virus, and Yellow fever virus [19,20,21]. While species such as *A. albopictus* typically require warm temperatures, *A. koreicus* and *A. japonicus* seem well adapted to more-temperate environments. *Aedes koreicus* prefers urban habitats and lays eggs in man-made containers, whereas *A. japonicus* prefers more sylvan and rural habitats, although it can tolerate urban habitats [22,23].

Available genome data and studies for *A. koreicus* are still fragmentary, mainly consisting of mitochondrial markers often analysed at a regional geographic scale [17,24]. A draft genome and mitogenome of an *A. koreicus* individual sampled from Hungary has been recently published [25], and two identical *A. koreicus* mitogenomes from Korea have been sequenced [26]. Although characterised by a larger invasive range, *A. japonicus* is poorly characterised from a molecular point of view; microsatellites and some mitochondrial markers are available [20,27,28,29,30], and a complete mitogenome sequence from a Hawaiian sample has been recently published [31]. More genome-scale data of both species from their invasive range are required to confidently infer their evolutionary history.

*Aedes koreicus* and *A. japonicus* are sister species (closest relatives in a phylogenetic tree) within a suggested species complex [14]. The timing of their origin is unclear as it is dataset dependent. Mitochondrial genes set the split between the two species at circa 46 Ma, nuclear genes at just circa 4 Ma, and a mixed dataset of nuclear and mitochondrial markers recovers intermediate estimates of circa 20 Ma [32,33]. The molecular systematics of these two species is also unclear. Current knowledge of *A. japonicus* systematics indicates the presence of four allopatric subspecies (*A. j. japonicus*, *A. j. yaeyamensis*, *A. j. amamiensis*, *A. j. shintiensis*), which show an overlap of morphological characteristics among them and with *A. koreicus*, which is often sympatric with *A. j. japonicus*. Molecular phylogenetic analyses [14] based on two mitochondrial (COII and fND4) and one nuclear (28S) marker suggest that genetic distances are compatible with the presence of four species. However, a comprehensive screening based on statistical analysis is still missing for this clade [13].

To aid genomic studies of *A. japonicus* and *A. koreicus* and to quickly provide genetic markers for phylogenetic and epidemiological studies, here we report the short-read, shallow genome sequencing of two individuals of both species sampled from northern Italy. Genome skimming [34], even in the case of small N50 and/or reduced coverage, allows the extraction of genes for mitogenomic [35] and phylogenomic [36] analyses. Our genome data allow us to identify up to 90% of BUSCO genes in *A. japonicus*, characterise the associated bacterial and viral content, and extract whole mitochondrial genomes. We used these data to update our knowledge of *A. japonicus* and *A. koreicus* evolution by estimating their divergence times using mitogenomics and by performing a COI barcode analysis of *Aedes*.

## 2. Methods

### 2.1. Sampling, Sequencing, and Assembly of A. japonicus and A. koreicus Genomes and Mitogenomes

*Aedes japonicus* specimens were reared at Fondazione Edmund Mach from samples that were field collected in the Friuli-Venezia-Giulia region in 2021 by the Istituto Zooprofilattico Sperimentale delle Venezie. *Aedes koreicus* specimens were reared in captivity at Fondazione Edmund Mach, from samples collected in the Trentino-Alto Adige region in 2020. To lower *A. koreicus* heterozygosity, we attempted to establish an inbred line. We isolated a male and a female as soon as they developed into adults, and placed them in separate cages. In the cage, we positioned cotton soaked with sugar solution (10%) and an ovitrap filled with water, with a blotting paper for the oviposition. A blood meal was provided to females by exposing a hand of one of the authors (NZ) every two days until oviposition. The breeding was successful for two generations; the third generation did not produce offspring. Therefore, we performed the DNA extraction on an *A. koreicus* pupa of the second generation. For both species, the rearing conditions were set as follows: temperature 23/26 °C, stable humidity to 70%, day–night cycle kept at 16 h light, 8 h dark, following the protocol in [37].

DNA extraction was performed using the nucleon-spin tissue extraction kit Qiagen, optimised for insect-DNA extraction. Extraction employed one pupa of each species. DNA yield was assessed with Qubit. We sequenced single individuals to reduce heterozygosity. After extraction, library preparation and sequencing were performed by the NGS facility of the University of Trento, using a NOVASEQ platform to obtain pair-end reads of 150 nt length each, with an average insert size of 650 nt. Raw reads were quality checked using fastQC and assembled using MaSuRCa version 4.0.5 [38] with k-mer size estimated during the procedure. As suggested by the MaSuRCA developers, we performed the assembly without any trimming steps (https://github.com/alekseyzimin/masurca#3-running-the-masurca-assembler, accessed on 1 January 2021). We employed default parameters, except for JF_SIZE, which was adjusted for the expected size of the genomes (circa 1.2 Gb from previous *Aedes* sequencing). De novo assembly quality was assessed using BUSCOv5.0 against Insecta datasets (insecta_odb10) [39,40].

To extract and assemble mitogenomes we employed total raw reads using both MitoZ 3.2 [41] and MitoS [42] by trimming adaptors and filter reads that did not correspond with the chosen clade.

### 2.2. Microbial and Viral Profiling

Raw reads were quality checked using FastQC and trimmed with Trimmomatic-0.39, with the option PE.fa:2:30:10:1:TRUE LEADING:3 TRAILING:3 SLIDINGWINDOW:4:5 MINLEN:25. A repeat FastQC on trimmed reads confirmed the effectiveness of trimming. To screen for the presence of bacteria, archaea and microeukaryotes, cleaned reads (paired and unpaired) were used as input of MetaPhlAn-4.0 [43], which was run using the mpa_vOct22_CHOCOPhlAnSGB_202212 database. As this version of the program has not yet been updated to profile viruses, we also employed the latest version which allows viruses profiling (MetaPhlAn-3.0; [44]) to check for their presence, with the option “--add-viruses” and using the mpa_v30_CHOCOPhlAn_201901 database (which is the latest version of the MetaPhlAn database that is compatible with this option). Relative abundance plots were obtained separately for bacteria and viruses using hclust2. The number of reads and the coverage of non-viral taxa (estimated on the basis of the MetaPhlAn-4 database of markers) were obtained using the option “-t rel_ab_w_read_stats”.

### 2.3. Phylogenomic Datasets

Raw data from genomic and transcriptomic sequencing available for Culicidae plus some Brachycera outgroups were downloaded from the SRA database (October 2021) using the SRA Toolkit version 2.1.11 [45]. We assembled genomic data using the de novo assembler MaSuRCa version 4.0.5 [38] with default parameters. Transcriptome data were assembled using Trinity de novo transcriptome assembler version 2.13.2 [46] and translated as proteome with the Transdecoder software v5.5.0 (Haas, BJ. https://github.com/TransDecoder/TransDecoder, accessed on 1 April 2022). We predicted the sample proteomes using Augustus version 3.2.3 [47] and conducted a BUSCO (v. 4.0.5) analysis on all proteomes [39,40] to estimate the completeness of our dataset. All single-copy BUSCO genes were then extracted, aligned using a MAFFT V. 7.503 [48], and trimmed with TrimAl software v. 1.2 [49], allowing up to 50% gaps. To check BUSCO orthogroups quality, we generated single-copy gene trees with IQTree 2.1.4 [50] and rooted them using the software newick-tool (https://github.com/xflouris/newick-tools, accessed on 1 April 2022). For each tree with the corresponding alignment, we calculated the distribution of branch lengths in the tree and listed ‘problematic taxa’ for which the branch length was more than twice the standard deviation from the average length. Genes with more than 40% of taxa considered as ‘problematic’ were removed. Sequences from the alignments were then renamed with the seq function from seqKit package [51] and concatenated with FASconCAT [52]. The resulting supermatrix was analysed using FastTree (v. 2.1.11) [53] with default parameters.

### 2.4. Mitogenomic Datasets and Molecular Clock Analyses

To build a mitogenomic dataset for phylogenetic and clock analyses, we downloaded all Aedini complete mitogenomes available at August 2022 on GenBank and added them to our newly assembled *A. koreicus* and *A. japonicus* mitogenomes. To obtain a dataset comparable with previous phylogenetic studies that investigated Culicidae evolution, we employed a taxon sampling that included various non-Aedini Culicinae and Anophelinae as in [33,54]. We further added representatives of the Culicidae outgroups (Chaboridae, Corethrellidae, Dixidea) and two Chironomidae sequences to break the otherwise long internal branch between the *Drosophila*–mosquitoes split and the diversification of the Culicoidea. From the GenBank file, we extracted t-RNAs, ribosomal, and protein-coding genes using custom scripts and aligned every gene independently using MAFFT [55]. We concatenated each gene with FASconCAT [52], obtaining a multi-gene concatenated alignment of 15,388 nucleotides for 67 species. The dataset is available in Supplementary Materials as “Aedes_mito.aln” in fasta format.

We used BEAST v2.6 [56] to estimate divergences under a GTR + G replacement model, defined by IQ-TREE v1.6 as the best-fitting model [57]. We employed a root prior based on the fruit fly–mosquito split, using a normal distribution with a mean set at 260 Ma and a 95% prior distribution between 296 and 238 Ma, as previously performed by other authors [58,59]. We employed minimum-calibration points for the diversification of Anophelinae and Culicinae, both at 34 Ma, compatible with the oldest fossils known for each group [59,60]. We added a minimum calibration of 99 Ma for the Culicidae, according to *Priscoculex burmanicus* [61]). There is no indication of whether this fossil belongs to the crown or to the stem Anophelinae; we preferred to be as conservative as possible and used this fossil to constrain the Anopheline–Culicinae split, which is the Culicidae crown. We ran all MCMC chains for 200,000,000 generations. We checked the actual convergence with Tracer1.7 [62], discarded 20% of the trees as burn-in, and summarised the Bayesian analyses using TreeAnnotator. We employed the Birth and Death model (BD) and a relaxed lognormal clock, as previously shown to be suitable for Aedini divergence studies [33,54]. Moreover, we tested for a putative-outgroup sampling effect by rerunning the analysis without the non-Culicidae Culicomorpha.

### 2.5. Barcoding

We sampled all available *Aedes* Cytochrome Oxidase Subunit I (COI) from GenBank (May 2022), including those extracted from our newly assembled mitogenome data, using BLASTn on the assemblies. This compilation yielded a dataset comprising 5389 *Aedes* COI sequences, which was aligned using MAFFT [55]. We explored the available literature and databases to find other markers to complement the analysis of COI, but could not find any other marker with a large sample density in databases for both *A. japonicus* and *A. koreicus*. To reduce the overall diversity in the alignment and obtain a barcode that is specific to *Aedes* clade B [32,33], we retained samples from *A. japonicus*, *A. koreicus,* and their most proximal 17 outgroup species, chosen by inspecting the phylogenetic tree of the 5389 samples’ dataset built using FastTree [53], with model GTR + G4 with default settings. Included outgroups are *A. chrysolineatus* Theobald, 1907; *A. elsiae* Barraud, 1923; *A. harveyi* Barraud, 1923; *A. notoscriptus* Skuse, 1889; *A. pseudotaeniatus* Giles, 1901; *A. togoi* Theobald, 1907; *Ochlerotatus atropalpus* Coquillett, 1902; *O. baisasi* Knight and Hull, 1951; *O. eatoni* Edwards, 1916; *O. epactius* Dyer and Knab, 1908; *O. euiris* Dyar, 1922; *O. mediovittatus* Coquillett, 1906; *O. portonovoensis* Tewari and Hiriyan, 1992; *O. wardi* Reinert, 1976; *Haemagogus equinus* Theobald, 1903; *H. janthinomys* Dyar, 1921; and *H. mesodentatusas* Komp and Kumm, 1938. Following this curation, we obtained a new dataset of 1446 samples (661 nt long). Because of the high heterogeneity of sequence length and to exclude any potential effect of sequence length on barcode analyses, we generated two subsets. The first, “Aedes_COI_long.aln”, retained sequences longer than 600 nucleotides and underwent trimming of 11 nucleotides at the 5′ end and 21 nucleotides at the 3′ end; it comprises 489 samples and spans 621 nucleotides. The second,”Aedes_COI_short.aln”, retained sequences longer than 300 nucleotides and underwent trimming of 260 nucleotides at the 5′ end and 99 nucleotides at the 3′ end; this subset consists of 817 samples and 299 nucleotides in length. Both datasets are available in the Supplementary Materials. We calculated a pairwise distance matrix to infer a barcoding gap using the DistanceCalculator class of the TreeConstruction module of Biopython with the “identity” model [63]. We used custom scripts (available at https://github.com/AleTatti/Barcoding-Analysis/blob/main/BarcodingGap_Analysis_v12.py) to plot the distribution of intraspecific and interspecific genetic distances of the 17 outgroups as a training set, excluding those involving *A. japonicus* and *A. koreicus* as a test set. We assessed the distribution without considering outliers, which was detected by employing an interquartile range (*IQR*) approach; pairwise genetic distances exceeding 1.5 times the *IQR* were flagged as outliers (Equation (1)).

(1) $$x_{i}>Q3+1.5\;IQR\;⋃\;x_{i}<Q1-1.5\;IQR$$

where *x_i_* are pairwise genetic distances, *Q*3 is the third quartile, and *Q*1 is the first quartile.

We plotted the distribution of the genetic distances between *A. koreicus* and *A. japonicus*, and the intraspecific distances of *A. koreicus* and *A. japonicus* against all the other species using matplotlib v.3.5.2 [64]. A maximum likelihood phylogenetic tree was constructed from the Aedes_COI_long alignment using IQ-TREE [57] and employing the GTR + F + I + G4 model, (as deducted using model selection m TEST) and 1000 bootstrap replicates (-bb 1000) to assess the tree’s robustness.

## 3. Results and Discussion

### 3.1. Genome Skimming of A. japonicus and A. koreicus

**Figure 2:**
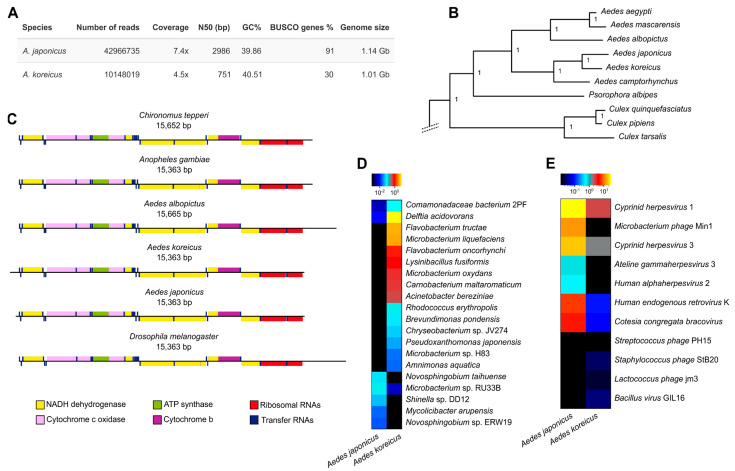
Genome skimming of *A. japonicus* and *A. koreicus*. (**A**) Short summary of sequence statistics (reads, coverage, N50, Busco values, genome length). (**B**) Phylogenetic position of the sequenced *A. japonicus* and *A. koreicus* according to a maximum likelihood phylogeny of BUSCO genes. Only the portion of the tree spanning *Aedes* species is depicted, while the full tree is in Supplementary Figure S2. (**C**) Structure of *A. japonicus* and *A. koreicus* mitogenomes compared with other dipteran species. (**D**) Bacterial profiling of the raw reads, with a heatmap showing the top 20 most abundant species. (**E**) Viral profiling of the raw reads, showing all the 11 viral species detected. The colours in the last two panels indicate the relative abundance of the bacterial/viral species detected in each of the two samples.

We obtained a total of 220 million reads for *A. japonicus* and 150 million reads for *A. koreicus*. The low average quality (Phred score) of the reverse reads of both species (Supplementary Table S1) forced the MaSuRCA assembler [38] to discard many reads, lowering the amount of total reads available for the assembly. The *A. japonicus* assembly resulted in an N50 of 2986, with an average coverage of 7.4X, whereas for *A. koreicus* the assembly provided a N50 of 765 bp, with an average coverage of 4.5X (Figure 2A). The poor assembly of *A. koreicus* is mirrored with the BUSCO assessment of gene completeness. While for *A. japonicus* we could retrieve 91% of genes, for *A. koreicus* we could find only 30% of mostly fragmented genes (Supplementary Figure S1). Genome data from both species are, however, enough for phylogenetic studies. *Aedes japonicus* provided a reasonable number of genes, indicating that this draft can be analysed in the future for the presence of genes belonging to gene families of ecological and management interest, such as opsin genes and odorant-receptors; phylogenomic studies of these gene families have been successfully performed using genomes of similar N50 and coverage [65,66]. We show that it is possible to use BUSCO genes to reconstruct a genome-scale phylogeny of the *A. japonicus* and *A. koreicus* (Figure 2B).

### 3.2. Aedes Mitogenomes Displayed a Deeply Conserved Structure

We reconstructed the complete mitogenomes of both species from a set of 60 million reads for *A. koreicus* and 75 million reads for *A. japonicus*, for coverage of at least 4000× and high-quality mitochondrial genomes [67]. The genome size is in line with that of other Aedini; gene content and order do not show any difference from other members of the Culicidae clade (Figure 2C). The overall genetic identity between *A. koreicus* and *A. japonicus* mitogenomes is 93%, whereas identity between *A. koreicus* from Italy and the *A. koreicus* mitogenome from Anyang-si, Republic of Korea (NC_046946) is 99.5%, based on 80 SNPs spread along the mitogenomes.

### 3.3. Different Microbial Profiles, High Presence of Delftia, and Absence of Wolbachia

As we sequenced from whole-body DNA extracts, many reads belong to other biological entities associated with mosquitoes. We quantified reads mapping to bacterial markers (see Section 2), detecting a low number of them for *A. japonicus* (*n* = 457,222, 0.32% of the total mapped reads; Supplementary Figure S3A) and a very high number in *A. koreicus* (*n* = 30,978,640, which corresponds to the 20.11% of total reads; Supplementary Figure S3B). The very high percentage of bacterial reads in *A. koreicus* suggests that the sample contained many contaminants, which may explain why the quality of this assembly was lower than that of *A. japonicus*. Our metagenomic mapping indicates that our two samples are characterised by distinct bacterial (Figure 2D and Supplementary Figure S3C) and viral (Figure 2E) content, although there are many similarities. In particular, bacterial taxa that were previously associated with *Aedes* mosquitoes, such as the Burkholderiales (which includes the Comamonadaceae, of which the genus *Delftia* is also part) and the Microbacteriaceae and Flavobacteriaceae families [68] were among the most abundant in both species (Supplementary Figure S3A–C). The difference between the abundances of Flavobacteriaceae and Sphingomonadaceae could be explained by different rearing conditions [69]. The detection of a highly abundant Comamonadaceae bacterium could indicate the presence of a symbiont interacting with the mosquitoes, which is interesting as members of the genus *Comamonas* have been found to influence egg production in *Aedes aegypti* and *Aedes atropalpus* [70]. The presence of *Delftia* is also noteworthy, as this symbiont has been shown to be able to reduce pathogen’s transmission in *Anopheles* mosquitoes [71], and its high relative abundance in both *A. japonicus* and *A. koreicus* could suggest a possible role in influencing vector biology of *Aedes* mosquitoes as well. We did not find evidence of the presence of the management-relevant endosymbionts such as *Wolbachia* and *Asaia*. This is congruent with previous analyses of mosquito microbiomes that found poor occurrence of *Wolbachia* in *A. koreicus* [68], and a high prevalence of this endosymbiont only in adult mosquitoes not in larvae and pupae [68,72,73,74], is consistent with our sampling from the pupae.

We detected more viruses in *A. japonicus* than in *A. koreicus* (Figure 2D), but this may be due to the sequencing depth, which was higher for *A. japonicus*. Interestingly, we found viruses associated with Microbacteriaceae (a bacterial taxon which is abundant in both species), such as *Microbacterium phage* and *Bacillus virus* GIL16. We detected *Cotesia congregata bracovirus*, which could suggest the presence of a similar virus associated with mosquitoes. While some detected bacteria and viruses can be robustly connected to mosquito biology, as discussed, others are likely contaminants from human handling (*Cutibacterium acnes*, *Human alphaherpesvirus* 2, *Human endogenous retrovirus* K, *Streptococcus phage* PH15, *Staphylococcus phage* StB20, *Ateline gammaherpesvirus* 3), or from the external environment (*Cyprinid herpesvirus* 1–3, *Lactococcus phage* jm3).

### 3.4. A More-Recent Timing of A. japonicus and A. koreicus Divergence Based on Mitogenomics

**Figure 3:**
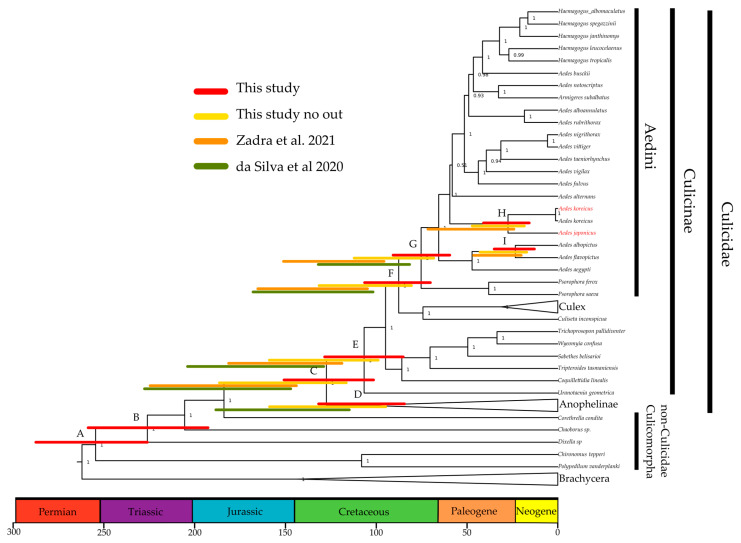
Divergence times of Aedini and other Culicidae mosquitoes. Posterior consensus tree from the analysis of the concatenated 13 coding proteins, 22 tRNAs and 2 rRNA genes of mitogenomes using nucleotides. Numbers at nodes are posterior probabilities and red bars are the 95% high posterior density (HPD) of estimates. Yellow bars are the HPD from the analysis of the same dataset using only the Culicidae taxa and no other Culicomorpha samples; orange and green bars indicate HPDs from previous studies (Zadra et al. (2021) [33]) and da Silva et al. (2020) [54], respectively. Details of the mean estimates and of the HPDs are presented in Table 1. The letters highlight node of interest that correspond to the one show in Table 1.

Previous phylogenies based on few marker genes found a discrepancy between mitochondrial and nuclear datasets for the divergence of *A. japonicus* from *A. koreicus* [33]. To test if this discrepancy is due to the use of few genetic markers, we inferred divergences from a new mitogenomic dataset that includes, for the first time, whole mitogenomes of *A. japonicus* and *A. koreicus (*mostly from our newly generated data). According to our calibrated Bayesian analysis (Figure 3 and Table 1), the split between *A. japonicus* and *A. koreicus* is circa 27 Ma (15–40 HPD%). This is a more-recent estimate than previously recovered [33] using four mitochondrial genes (mean 46 Ma), but still much older than the estimate from four nuclear genes (mean 4 Ma); this suggests that the amount of data used (and the type of outgroups, see below) is only partially responsible for the observed discrepancy between mitochondrial and nuclear data. Future studies should directly compare divergence estimates from mitogenomic datasets with phylogenomic (i.e., nuclear genome scaled) datasets.

Our estimated divergence falls within the Oligocene/Miocene epochs, which were marked by a prevalent cooling trend and the geological events that contributed to the formation of the Japanese archipelago [75,76,77]. Both *Ae. japonicus* and *Ae. koreicus* exhibit adaptation to temperate climates and are susceptible to developing in warm-to-hot temperatures (>30 °C) [37,78,79]. Consequently, we hypothesise that the common ancestor of these species evolved to endure cooler conditions, possibly influenced by the prevailing cooling trend of that period, and subsequent geological events likely played a role in the divergence of these two mosquito species.

**Table 1:** Divergence estimates of selected nodes from Figure 3 and other analyses. For each node, the mean and the 95% high posterior density is provided. Results of our study are compared with those of Lorenz et al. (2021), which did not provide HPD 95% estimates. All the estimates are provided in million years ago (Ma). **^1^** [33] ^2^ [54] ^3^ [80].

Node	Taxonomic Level	This Study	This Study Only Culicidae	Zadra et al. (2021) ^1^	da Silva et al. (2020) ^2^	Lorenz et al. (2021) ^3^
A	Culicomorpha	255 (226–287)				
B	Culicoidea	226 (192–258)				220
C	Culicidae	127 (101–150)	151 (115–186)	182 (143–223)	182 (146–233)	197
D	Anophelinae	106 (84–132)	125 (94–159)		145 (114–187)	147
E	Culicinae	106 (85–128)	128 (98–158)	150 (118–184)	160 (128–205)	153
F	Culicini–Aedini split	87 (70–106)	105 (79–131)	135 (104–164)	130 (101–168)	123
G	Aedini	75 (59–90)	90 (67–113)	111 (95–150)	102 (81–132)	74
I	*A. albopictus–A. flavipictus*	23 (12–34)	29 (16–43)	33 (20–46)		
H	*A. koreicus–A. japonicus*	27 (15–40)	32 (18–48)	46 (24–71)		

### 3.5. Outgroup-Rich Phylogenomics Provide a More-Recent Picture of Mosquito Radiations

Our dated phylogeny of mosquitoes is topologically congruent with previous studies, but differs in the timescale of mosquito evolution. Our analysis (95% High Posterior Densities-HPD as red bars in Figure 3) consistently provides more-recent divergences compared to previous estimates (orange and green bars, see also Table 1). For example, the diversification of *Aedes* (clade A and B split [32,33], node G) has a mean of 75 Ma (HPD 95%: 59–90) in the current analysis, while it was older in analyses of partial-genome mitochondrial data, with means of 102 Ma and 111 Ma in [54] and [33], respectively. The diversification of Anopheline (Node D) and Culicinae (Node E) took place almost simultaneously circa 106 Ma, up to 50 Ma more recently than previously suggested; the split between Culicini and Aedini (Node F) occurred circa 87 Ma (HPD 95%: 70–106) in our analysis, while previous estimates propose this divergence at circa 130 Ma [33,54,80]. Probably the most relevant difference between previous works and here is for the Culicidae split (node C); our 95% HPD estimates barely overlap with previous ones [32,33,54,81].

The more-recent divergences retrieved here can be explained using a large outgroup sampling, which may have broken the otherwise long branch that leads from the root to the Culicidae group. The ingroup and outgroup sampling have been previously shown to affect the divergence estimates in Bayesian frameworks [82,83,84]. To test if the outgroup choice affects tree topology and posterior distribution, we ran the analysis without the non-Culicidae Culicomorpha species (*Polypedilum vanderplanki*, *Chironomus tepperi*, *Dixella* sp., *Corethrella condita,* and *Chaborus* sp.). In the absence of these outgroups, the mean estimates become older (Table 1), and the 95% HPD (yellow bars in Figure 3) becomes wider for the diversification of all clades. This indicates that including non-Culicidae Culicomorpha outgroup increases the precision of the Culicidae estimates. The discrepancies between our and other studies can also be explained using the dataset employed. Our analysis employed all genes, whereas others used only the protein-coding genes [54] or only limited mitochondrial markers [33]. Another possible source of discrepancy is taxon sampling within Culicidae. Da Silva et al. (2021) employed, for example, more *Anopheles* and less Aedini than us, something which may have affected the distribution of inferred ages of ancestral nodes.

### 3.6. A Large COI Barcode Analysis Supports Multiple Species within an Aedes japonicus + Aedes koreicus Species Complex

Previous phylogenetic investigation of *A. japonicus* and *A. koreicus* systematics, using the mitochondrial markers COII and ND4, retrieved *A. koreicus* nested within a paraphyletic *A. japonicus* composed of the subspecies *A. j. japonicus*, *A. j. yaeyamensis*, and *A. j. amamiensis* [14]. The morphological characters employed for distinguishing the four putative *A. japonicus* subspecies and *A. koreicus* largely overlap, suggestive of a species complex and could lead to possible misidentification of adults [14].

**Figure 4:**
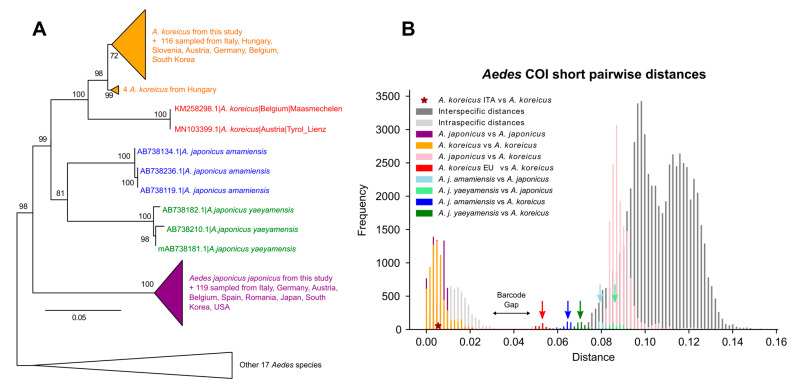
COI barcoding analyses of *A. japonicus* and *A. koreicus*. (**A**) Maximum likelihood phylogeny of various *Aedes,* based on COI marker. Triangles indicate a collapsed clade. Numbers at nodes represent bootstrap supports. Note that *A. koreicus* (in orange) is nested within paraphyletic *A. japonicus*, composed of three distinct groups: *A. j. japonicus* (purple), *A. j. yaeyamensis* (green), and *A. j. amamiensis* (blue). Two *A. koreicus* samples from Austria and Belgium (in red) exhibit genetic distances compatible with a distinct clade. (**B**) Barcoding gap analysis using COI marker. Different colours indicate pairwise distance between different groups. Note that the distribution of interspecific distances (on the right of the barcode gap) includes distances between *A. j. japonicus* and *A. j. amamiensis* or *A. j. yaeyamensis* (respectively, light blue and light green bars and arrow). The lower tail of the interspecific distribution includes distances between two samples of Austrian and Belgian *A. koreicus* against all other *A. koreicus* (red).

To further test the possibility of a species complex, and to test the taxonomic status of the individuals that we sequenced, we computed a large COI phylogenetic and barcoding gap analysis based on 491 COI sequences from *A. japonicus*, *A. koreicus*, and their closest outgroups (*Aedes* species belonging to clade B, see methods). The COI phylogenetic tree (Figure 4A) suggests that *A. koreicus* is nested within a paraphyletic *A. japonicus* composed of three extremely distinct groups: *A. j. japonicus*, *A. j. yaeyamensis*, and *A. j. amamiensis.* This is in accordance with the previous study outlined above [14] and the COI barcoding gap analysis of Figure 4B. The reciprocal distances between *A. j. japonicus*, *A. j. yaeyamensis*, and *A. j. amamiensis* (light blue and light green arrows) clearly fall outside the barcoding gap and within inter-specific distributions, indicating the presence of three distinct species. These distances are greater than those between *A. korecius* and *A. j. yaeyamensis or A. j. amamiensis* (blue and green arrows), indicating that *A. j. yaeyamensis* and *A. j. amamiensis* are closer related to *A. koreicus* than to *A. j. japonicus.* Our barcode analyses are overall compatible with the presence of at least three distinct species rather than three subspecies within a species complex. We formerly suggested naming them as *A. japonicus*, *A. yaeyamensis,* and *A. amamiensis.* Previous studies demonstrated mating incompatibilities between *A. koreicus* and *A. j. japonicus* [85], and future studies should focus on performing mating experiments between *A. japonicus*, *A. yaeyamensis,* and *A. amamiensis.*

We further identified two nearly identical *A. koreicus* samples from Austria and Belgium whose distance from other *A. koreicus* is compatible with a distinct species (red arrow). This new species is clearly closely related to *A. koreicus*, but according to the barcode gap of Figure 4B should be considered a separate, still uncharacterized species: this is in accordance with a previous study [86] that could not assign these two samples to *A. koreicus* using a barcode gap approach. More data from the Asian native range and newly introduced countries (for example, from the two samples identified as a new species sister to *A. koreicus*) is needed to better understand the reciprocal affinities of *A. japonicus* and *A. koreicus.*

## 4. Conclusions

In this study, we generated short-read draft genomes from Italian individuals of *A. japonicus* and *A. koreicus*. We used this resource for genome skimming to improve knowledge of the evolution of these two species. Our divergence estimates based on whole mitogenomes recover the origin of these two species at circa 27 Ma, and more recently than that previously reported using mitochondrial data. Our estimates of Aedini evolution are in general more recent than those previously reported, likely because we employed a larger outgroup sampling for the mosquito clade. Our COI barcoding and phylogenetic analyses indicate that *A. japonicus yaeyamensis*, *A. japonicus amamiensis*, and two *A. koreicus* sampled from Europe should be considered separate species. Overall, our phylogenetic and barcode studies advance our understanding of *A. japonicus* and *A. koreicus* evolution and demonstrate the need to obtain whole-genome data from the three potential new species we identify, in order to further disentangle their complex patterns of evolution.

## Data Availability

Mitogenomes and raw data are deposited in NCBI, mitogenome Accession: OR668893, OR668894. Raw data: PRJNA1027810. Alignments used are available as supplementary data.

## Supplementary Materials

The following supporting information can be downloaded at https://www.mdpi.com/article/10.3390/insects14120904/s1. Supplementary figures and materials are available online. Figure S1: Supplementary Figure S1; Figure S2: Supplementary Figure S2; Figure S3: Supplementary Figure S3; Table S1: Supplementary Table S1.docx. In the supplementary material we provide all the figure of the paper in three different format: png, pdf and svg.
