# Supplementary material for: Shallow Whole-Genome Sequencing of Aedes japonicus and Aedes koreicus from Italy and an Updated Picture of Their Evolution Based on Mitogenomics and Barcoding
Source: Insects. 2023 Nov 23;14(12):904. doi: 10.3390/insects14120904 (PMC10743467; doi:10.3390/insects14120904)

A

Species	Number of reads	Coverage	N50 (bp)	GC%	BUSCO genes %	Genome size
A. japonicus	42966735	7.4x	2986	39.86	91	1.14 Gb
A. koreicus	10148019	4.5x	751	40.51	30	1.01 Gb

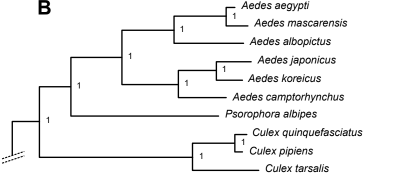

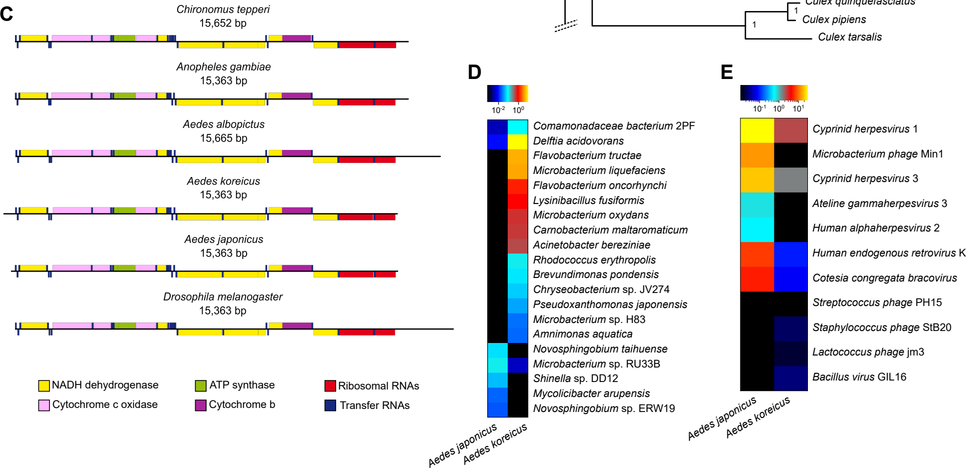

Supplement: Supplementary file 1 [file insects-14-00904-s001.zip › Supplementary/High_quality_fig_and_supp/Figure2.pdf]
