# Supplementary material for: Shallow Whole-Genome Sequencing of Aedes japonicus and Aedes koreicus from Italy and an Updated Picture of Their Evolution Based on Mitogenomics and Barcoding
Source: Insects. 2023 Nov 23;14(12):904. doi: 10.3390/insects14120904 (PMC10743467; doi:10.3390/insects14120904)
Supplement: Supplementary file 1 [file insects-14-00904-s001.zip › Supplementary/High_quality_fig_and_supp/SupplementaryFigure1.pdf]

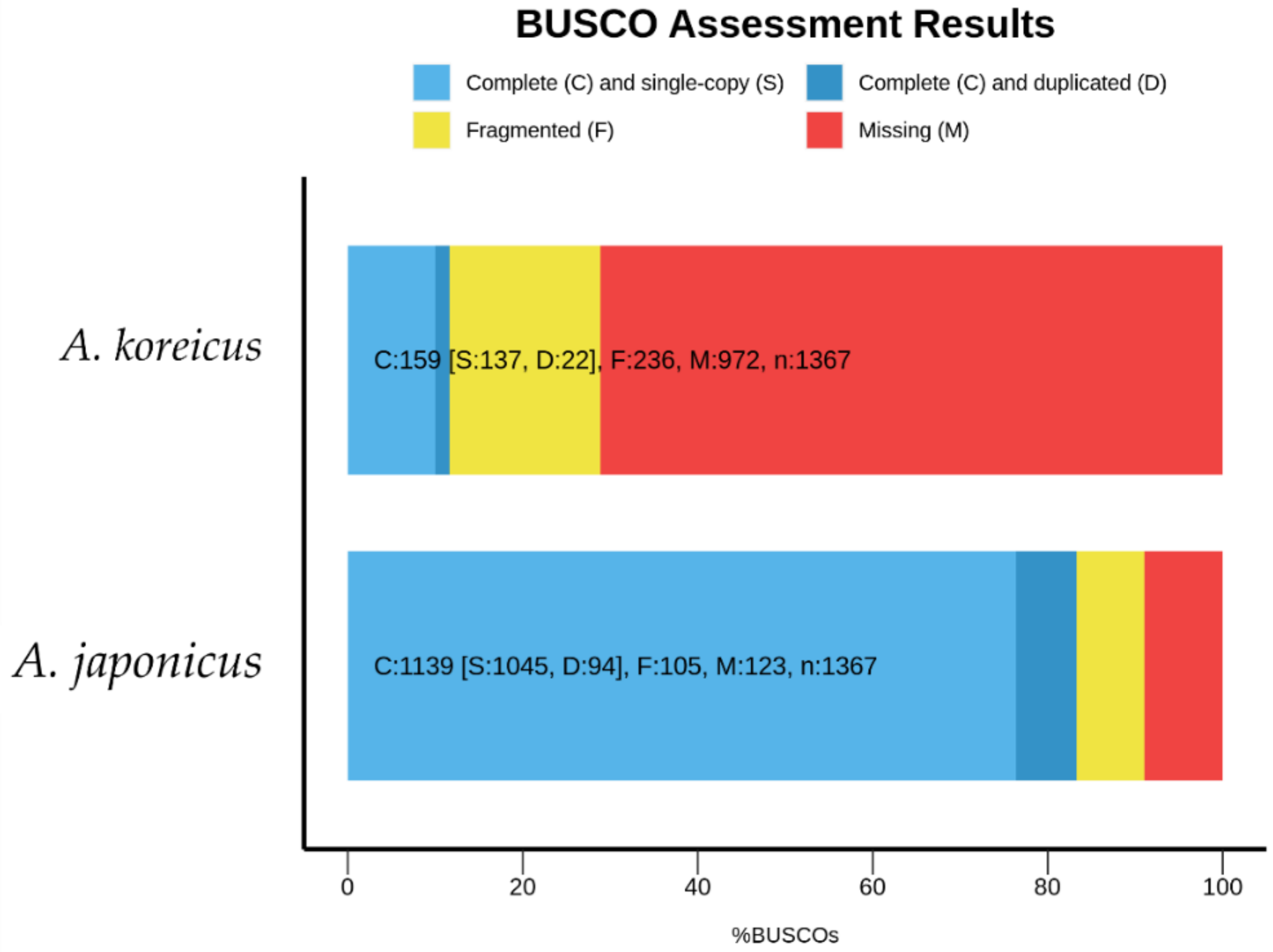

Supplementary figure 1. BUSCO assessment of the genome assemblies. The analysis was carried out employing the Insect single-copy orthologous dataset (insecta_odb10). The light blue bar indicates the percentage of complete genes found by BUSCO in single copy; the dark blue bar represents the duplicated complete genes found; the yellow bar shows the percentage of fragmented genes; eventually, the red bar indicates the missing genes, namely the genes that are supposed to be present in the species in question but not found in the genome assembly provided to BUSCO.
