# Supplementary material for: Shallow Whole-Genome Sequencing of Aedes japonicus and Aedes koreicus from Italy and an Updated Picture of Their Evolution Based on Mitogenomics and Barcoding
Source: Insects. 2023 Nov 23;14(12):904. doi: 10.3390/insects14120904 (PMC10743467; doi:10.3390/insects14120904)
Supplement: Supplementary file 1 [file insects-14-00904-s001.zip › Supplementary/SupplementaryTable1.pdf]

Supplementary Text

	Reads	N° sequence	Duplicates (%)	Quality score
**A. koreicus**	R1	79.4	11.0	35.5
	R2	79.4	33.9	27.3
**A. japonicus**	R1	110.7	14.1	35.2
	R2	110.7	26.5	29

Supplementary Table S1. The reads quality check obtained with fastqc, shows high duplicated rates in both, especially in *A. koreicus*, moreover, the amount of reads in *A. koreicus* is substantially lower than in *A. japonicus*. The quality scores show that probably there was a problem during the sequencing of the reverse strand of the pair-end reads.
