# Supplementary figures and images for: Shallow Whole-Genome Sequencing of Aedes japonicus and Aedes koreicus from Italy and an Updated Picture of Their Evolution Based on Mitogenomics and Barcoding
Source: Insects. 2023 Nov 23;14(12):904. doi: 10.3390/insects14120904 (PMC10743467; doi:10.3390/insects14120904)

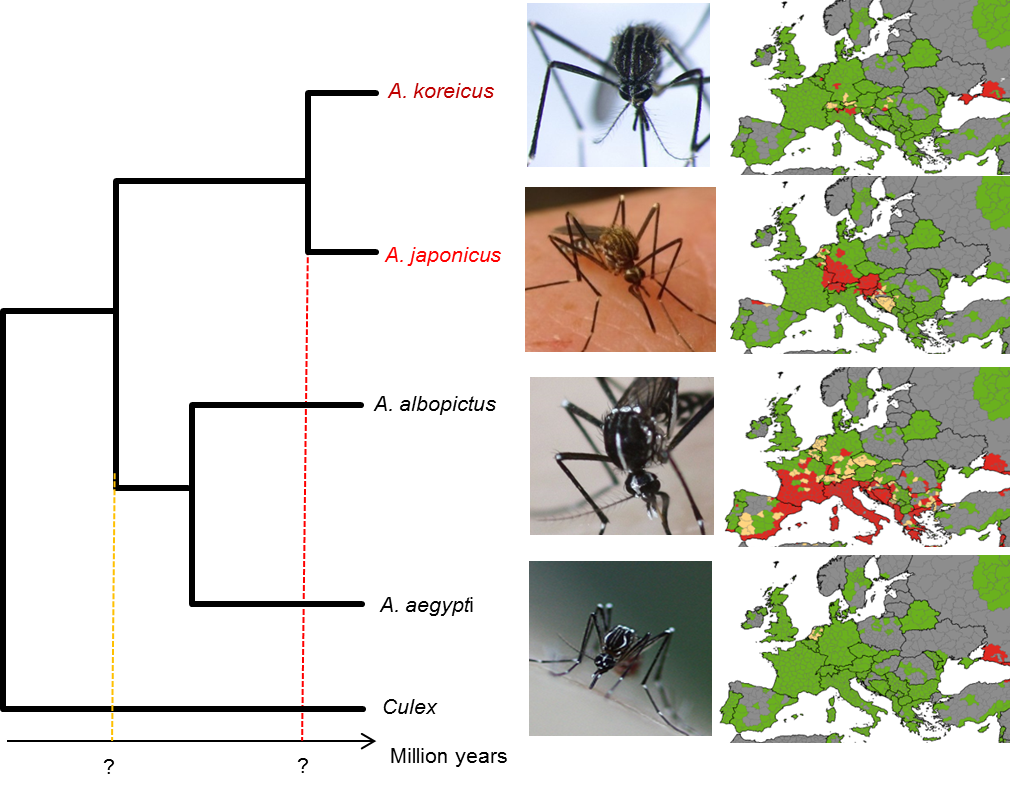

Supplement: Supplementary file 1 [file insects-14-00904-s001.zip › Supplementary/High_quality_fig_and_supp/Figure1.png]

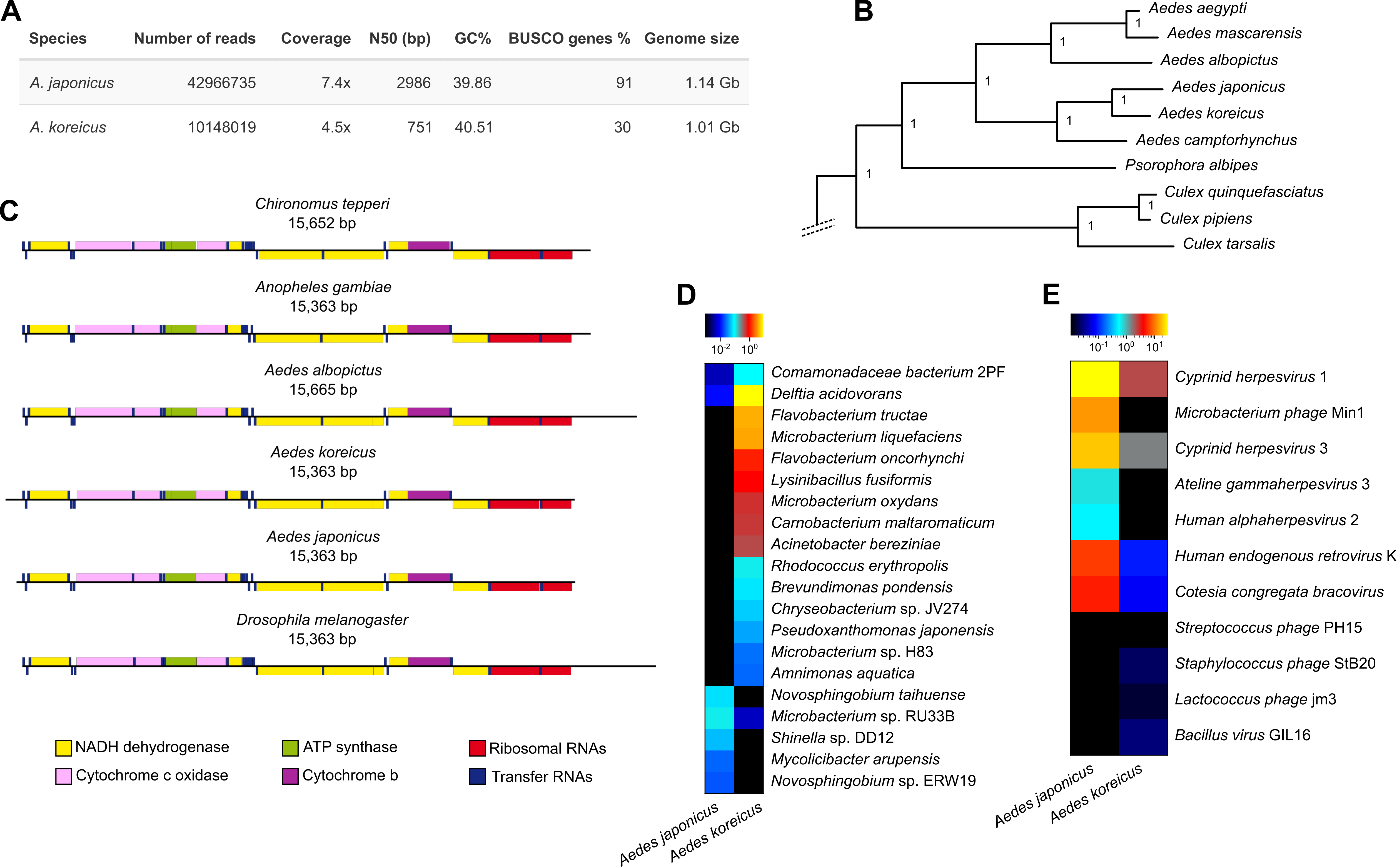

Supplement: Supplementary file 1 [file insects-14-00904-s001.zip › Supplementary/High_quality_fig_and_supp/Figure2.png]

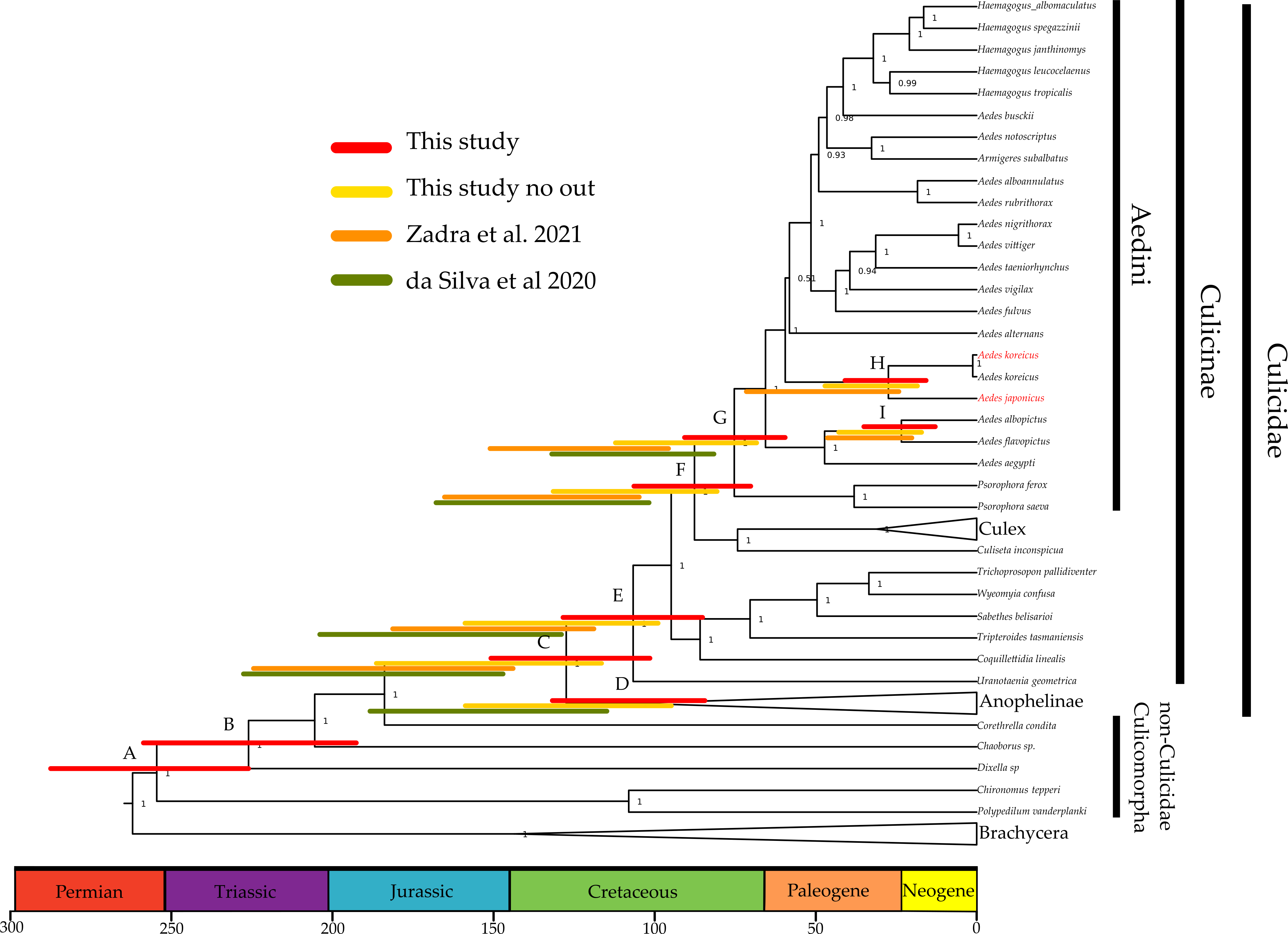

Supplement: Supplementary file 1 [file insects-14-00904-s001.zip › Supplementary/High_quality_fig_and_supp/Figure3.png]

**A**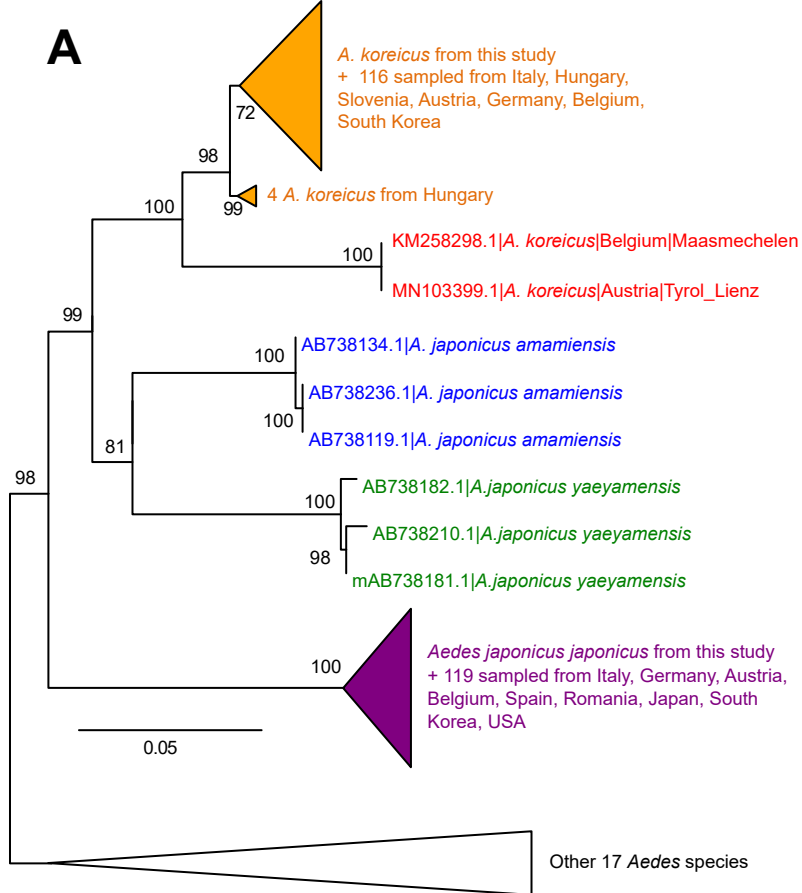**B**

### *Aedes* COI short pairwise distances

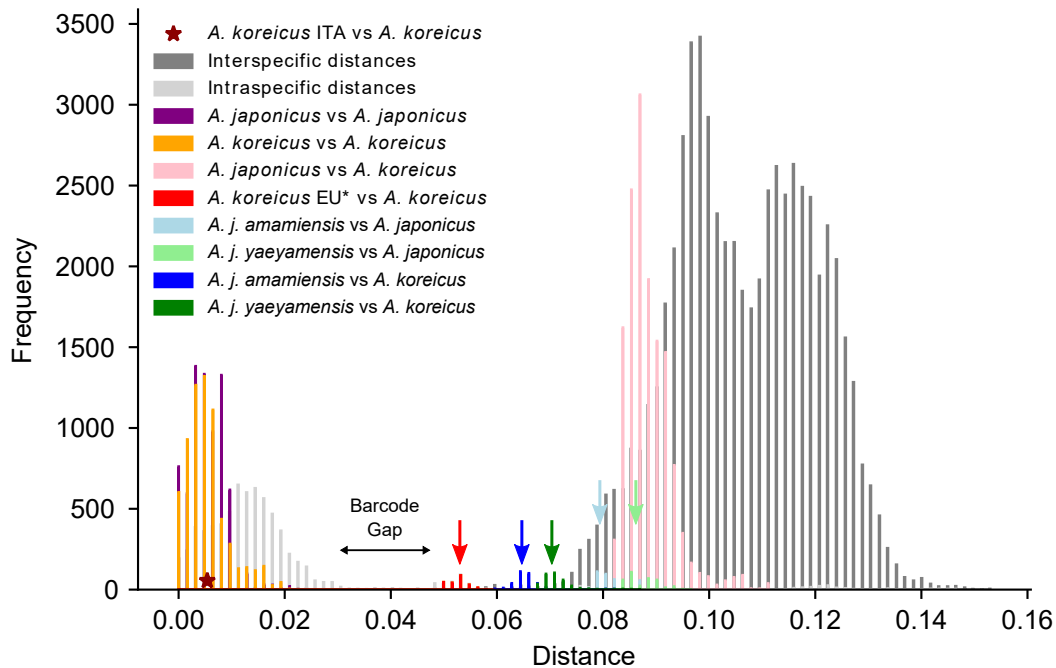

Supplement: Supplementary file 1 [file insects-14-00904-s001.zip › Supplementary/High_quality_fig_and_supp/Figure4.pdf]

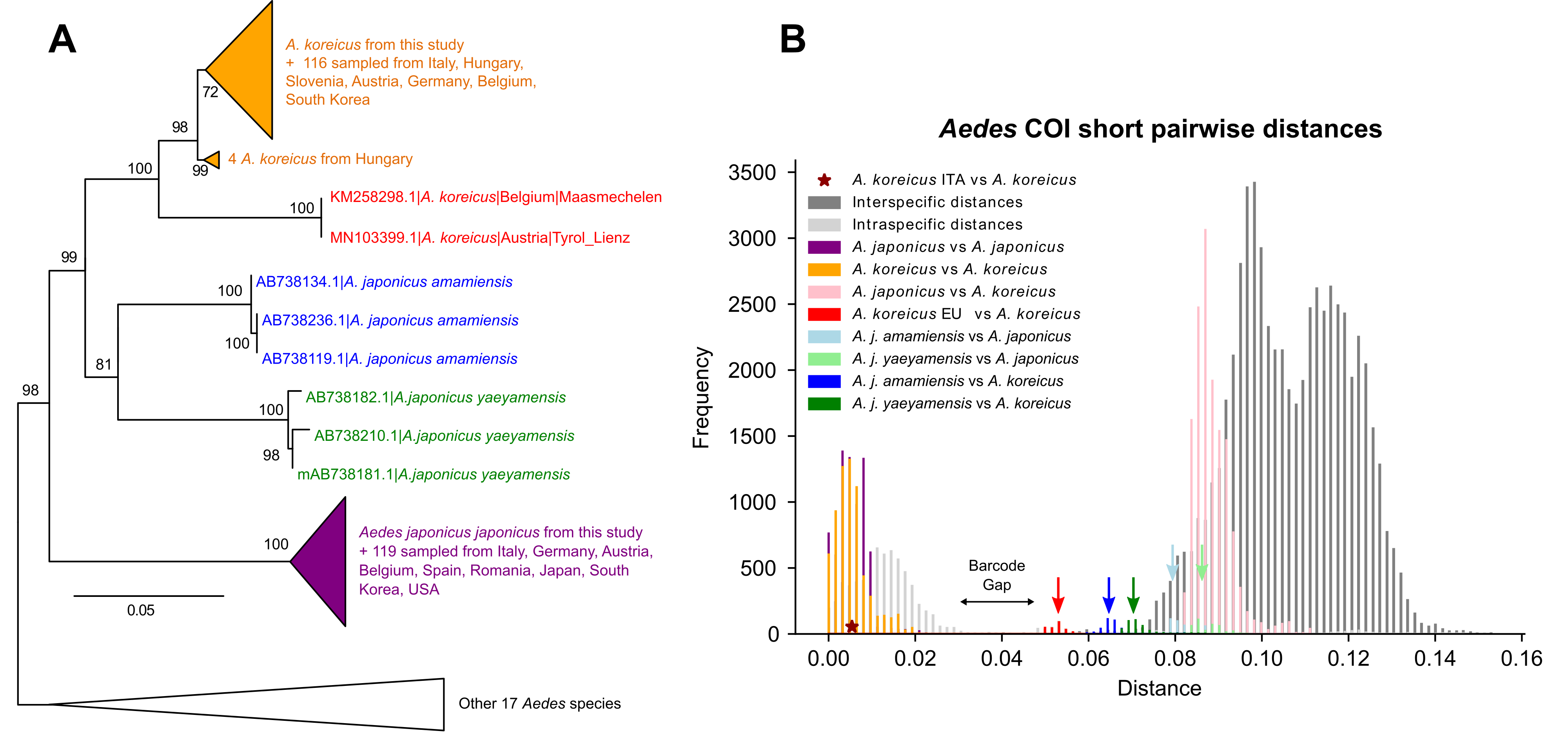

Supplement: Supplementary file 1 [file insects-14-00904-s001.zip › Supplementary/High_quality_fig_and_supp/Figure4.png]

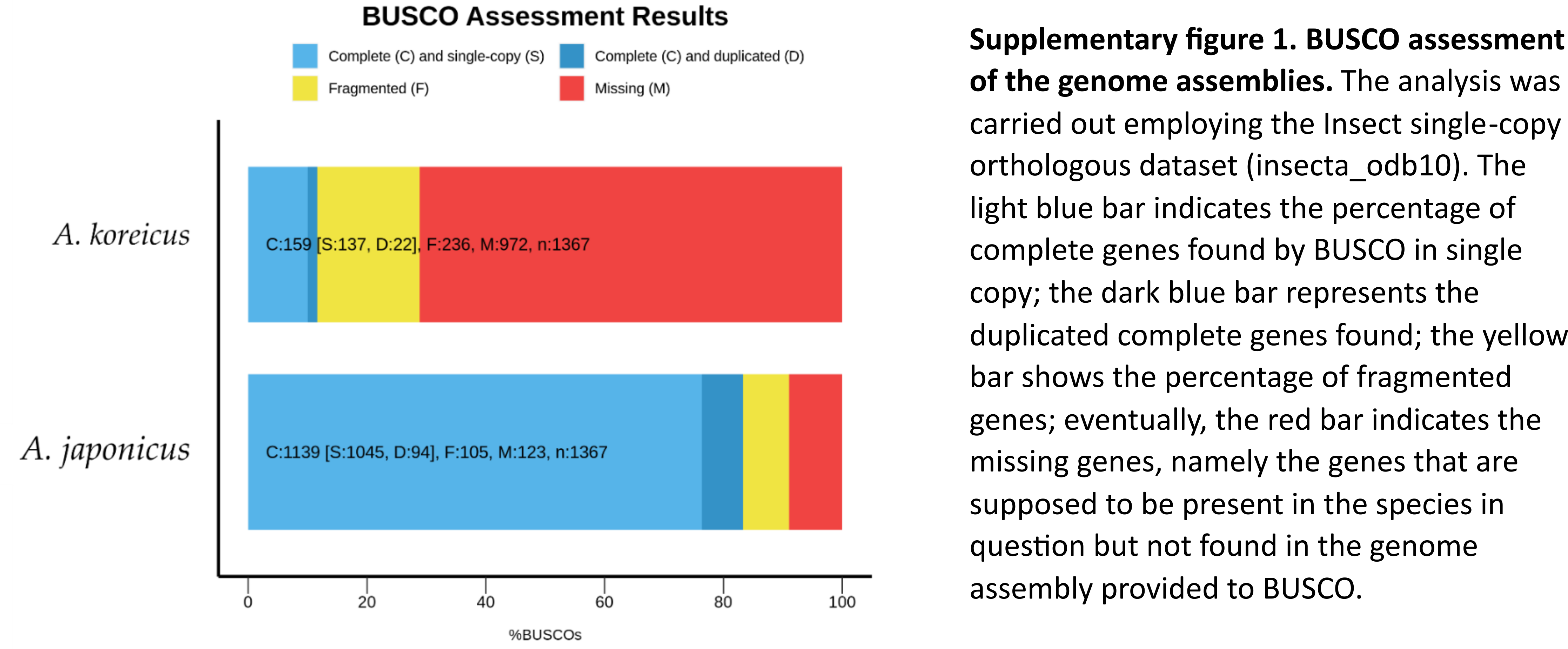

Supplement: Supplementary file 1 [file insects-14-00904-s001.zip › Supplementary/High_quality_fig_and_supp/SupplementaryFigure1.png]

# Nuc\_aa fasttree

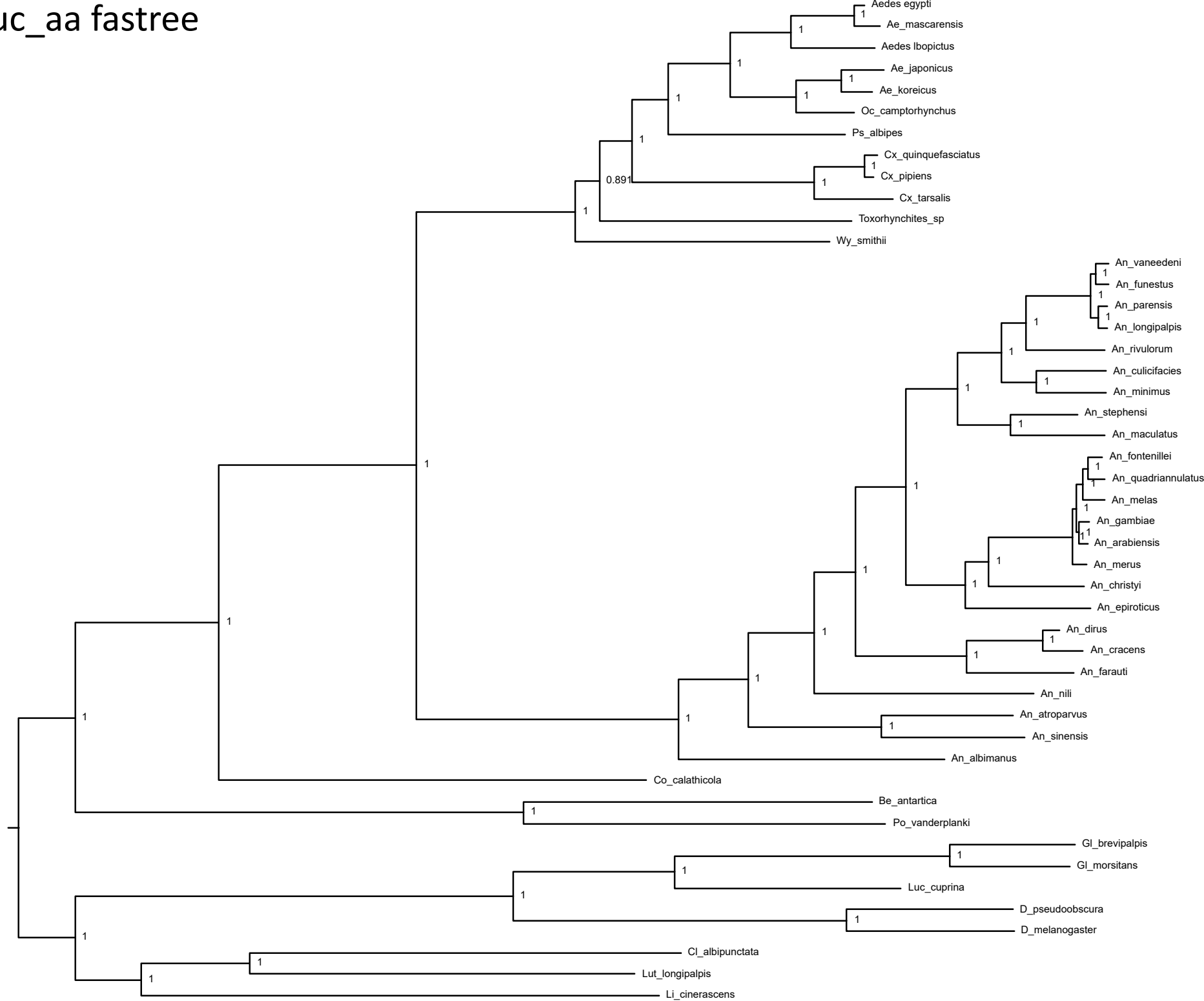

0.2

Supplement: Supplementary file 1 [file insects-14-00904-s001.zip › Supplementary/High_quality_fig_and_supp/SupplementaryFigure2.pdf]

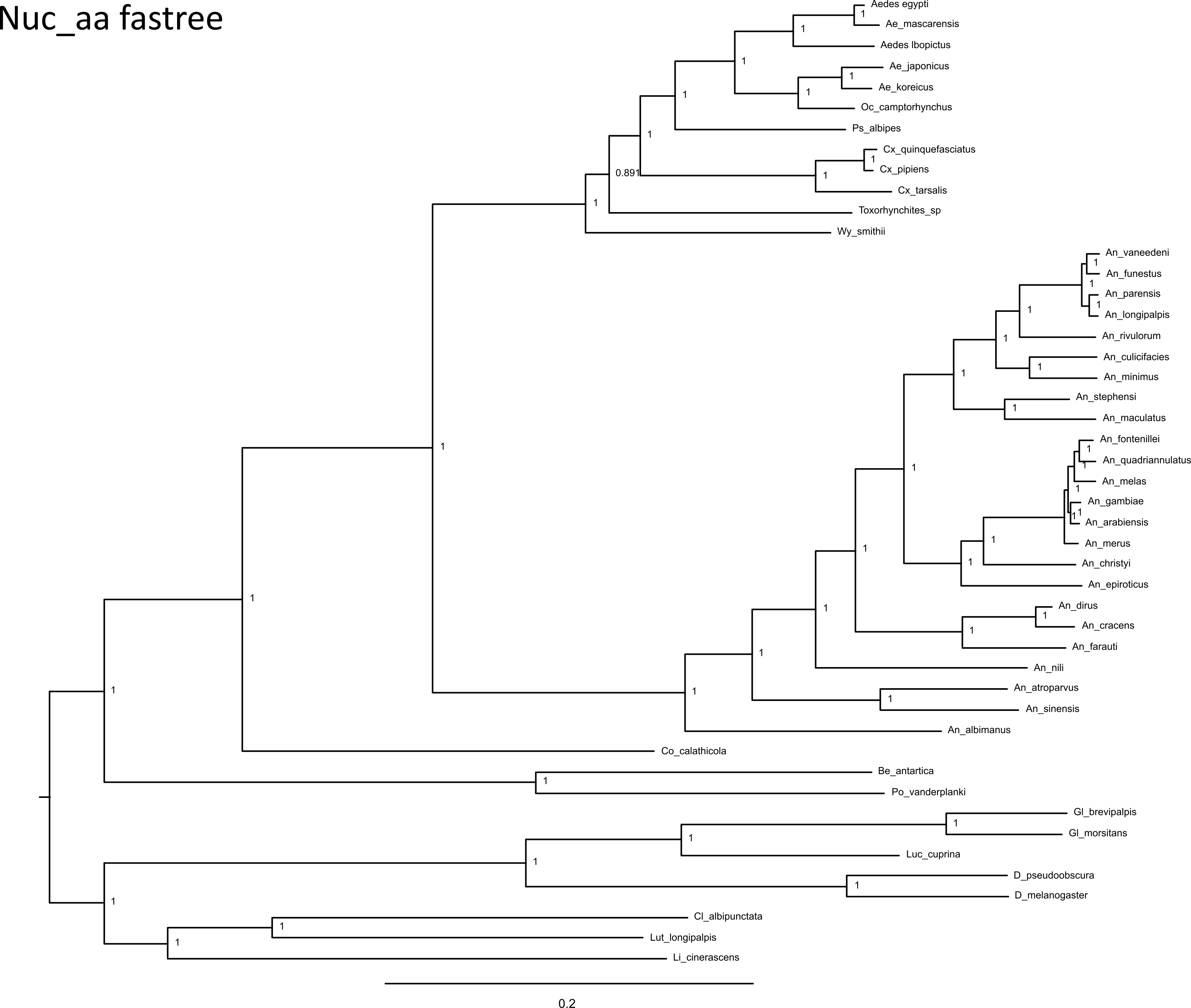

Supplement: Supplementary file 1 [file insects-14-00904-s001.zip › Supplementary/High_quality_fig_and_supp/SupplementaryFigure2.png]

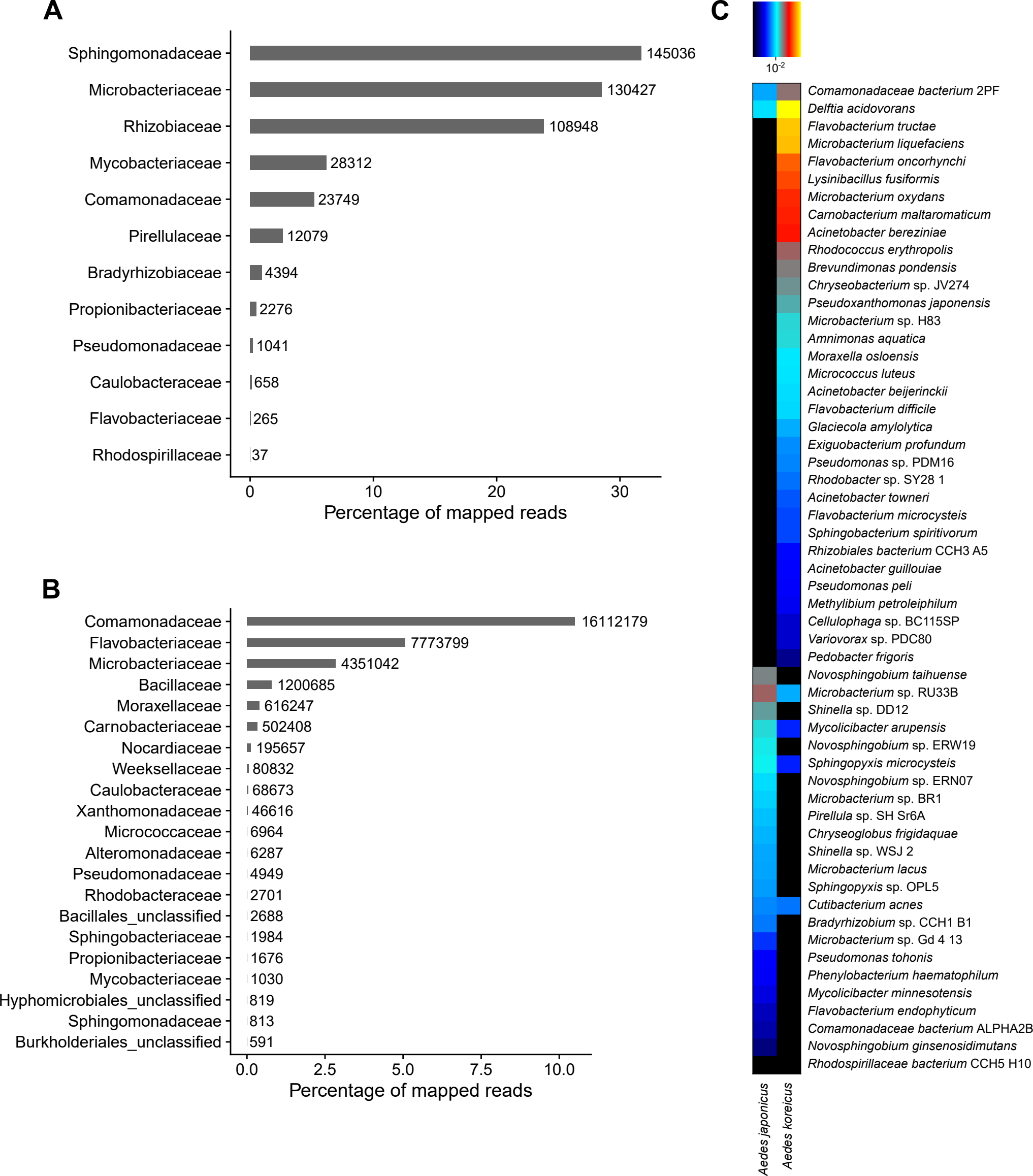

Supplement: Supplementary file 1 [file insects-14-00904-s001.zip › Supplementary/High_quality_fig_and_supp/SupplementaryFigure3.pdf]
